# Auscultation and Percussion

**Published:** 1883-07

**Authors:** 


*Auscultation and Percussion.* By SAMUEL GEE, M.D. Third Edition, pp. 344. London: Smith, Elder & Co., 1883.

This work we consider to be one of the very best on the methods of physical examination of the chest. Great care has evidently been taken in the arrangement of the material and the description of the methods. Everything is elaborately tabulated and accurately headed; and the most perfect system and regularity obtains throughout. In the allotment of space special regard has been had to the practical value of the various methods, and those means of examination which are only of theoretical interest are not dwelt upon. The character of the style, a most important item in a work of this sort, is worthy of all praise, being clear, concise and vigorous. We heartily recommend this work as a model of its kind, valuable equally to student and to practitioner.

